# The Different Mechanisms of Lipid Accumulation in Hepatocytes Induced by Oleic Acid/Palmitic Acid and High-Fat Diet

**DOI:** 10.3390/molecules28186714

**Published:** 2023-09-20

**Authors:** Miao Zhang, Xue Bai, Qian Du, Jiaojiao Xu, Danqing Wang, Lei Chen, Keting Dong, Ziyue Chen, Jianhong Yang

**Affiliations:** 1 Savaid Medical School, University of Chinese Academy of Sciences, Beijing 101400, China; zhangmiao21@mails.ucas.ac.cn (M.Z.); paihsue@foxmail.com (X.B.); cathydu0827@126.com (Q.D.); 15733156243@163.com (J.X.); wangdanqing19@mails.ucas.ac.cn (D.W.); chenlei20@mails.ucas.ac.cn (L.C.); dongketing21@mails.ucas.ac.cn (K.D.); 2 School of Nursing, Capital Medical University, Beijing 100069, China; chen15560086071@163.com

**Keywords:** NAFLD, lipid accumulation, cellular models, animal models, oleic acid and palmitic acid, HFD, de novo lipogenesis, fatty acid uptake, FASN, CD36

## Abstract

Non-alcoholic fatty liver disease (NAFLD) is the primary chronic liver disease worldwide, mainly manifested by hepatic steatosis. Hepatic lipids may be derived from dietary intake, plasma free fatty acid (FFA) uptake, or hepatic de novo lipogenesis (DNL). Currently, cellular and animal models of hepatocellular steatosis are widely used to study the pathogenesis of NAFLD and to investigate therapeutic agents. However, whether there are differences between the in vivo and in vitro models of the mechanisms that cause lipid accumulation has not been reported. We used OA/PA-induced NCTC 1469 cells and high-fat-diet-fed C57BL/6J mice to simulate a hepatocyte steatosis model of NAFLD and to detect indicators related to FFA uptake and DNL. In addition, when serological indicators were analysed in the mouse model, it was found that serum FASN levels decreased. The results revealed that, in the cellular model, indicators related to DNL were decreased, FASN enzyme activity was unchanged, and indicators related to FFA uptake were increased, including the high expression of CD36; while, in the animal model, indicators related to both FFA uptake and de novo synthesis were increased, including the high expression of CD36 and the increased protein levels of FASN with enhanced enzyme activity. In addition, after an analysis of the serological indicators in the mouse model, it was found that the serum levels of FASN were reduced. In conclusion, the OA/PA-induced cellular model can be used to study the mechanism of FFA uptake, whereas the high-fat-diet-induced mouse model can be used to study the mechanism of FFA uptake and DNL. Combined treatment with CD36 and FASN may be more effective against NAFLD. FASN in the serum can be used as one of the indicators for the clinical diagnosis of NAFLD.

## 1. Introduction

Non-alcoholic fatty liver disease (NAFLD) is characterised by hepatocyte steatosis and lipid accumulation caused by factors other than the exact inducing factors such as alcohol. Over the past four decades, NAFLD has become the most common chronic liver disorder (with a global prevalence of approximately 25% of the adult population) [1], and this disease can progress to cirrhosis, liver failure, and hepatocellular carcinoma, posing a threat to human health [2]. Therefore, knowledge about the pathophysiology and therapeutic factors of NAFLD could help clinicians to better prevent, monitor, and treat the disease.

In studying the pathogenesis of NAFLD, ethical issues, the limitations of tissue biopsy, and difficulties in obtaining human tissue samples have led to the need to find alternative research subjects. In addition, the advantages of cellular models of human disease are that they simulate the characteristic pathological changes in cells, are easy to manipulate, and are reproducible. The advantage of animal models of human disease is that animals can be used to simulate experiments, strictly control conditions, eliminate confounding factors, and avoid harm to the human body. Therefore, cellular models and animal models are often used as alternative subjects to study the mechanisms underlying various diseases.

It is well-known that sugars and lipids play an important role in cellular composition and life activities. However, the excessive intake of sugars and lipids leads to disease. Under physiological conditions, the sources and destinations of fatty acids in the liver are in a dynamic balance. When this balance is disturbed, it leads to disturbances in the fatty acid metabolism, including lipid accumulation. With changes in people’s diets, an excessive carbohydrate intake can lead to disturbances in the energy metabolism of hepatocytes and, thus, an increase in the sources of fatty acids. Therefore, it is challenging to establish a suitable cellular model and animal model to study the mechanisms of cellular steatosis. In the construction of cellular steatosis models, fatty acids are usually used as inducers. At present, oleic acid (OA) [3,4], palmitic acid (PA) [5,6], and OA/PA [7,8] are mainly used to induce hepatocyte steatosis. These models were also widely used to study the mechanism of cellular steatosis. In the construction of animal models of NAFLD, the dietary induction most closely resembles that of the human NAFLD situation, of which high-fat diets are the most common [9,10]. It is now accepted that HFD (45–75% of the total caloric intake from fat) can be used to induce metabolic syndrome, hepatic steatosis, and NASH in experimental animals [11]. However, whether the mechanisms of lipid accumulation are consistent between cellular and animal models has not been reported.

In this study, we established a model of the OA/PA-induced lipid accumulation in hepatocytes and a model of HFD-induced NAFLD and investigated the main causes of the lipid accumulation in these models. In addition, we compared the serum indicators in the mouse model of NAFLD and in C57BL/6J mice to provide new insights for NAFLD mechanism studies and clinical drug trials.

## 2. Results

### 2.1. Establishment of a Cell Model of Lipid Accumulation Induced by OA/PA

**Figure 1:**
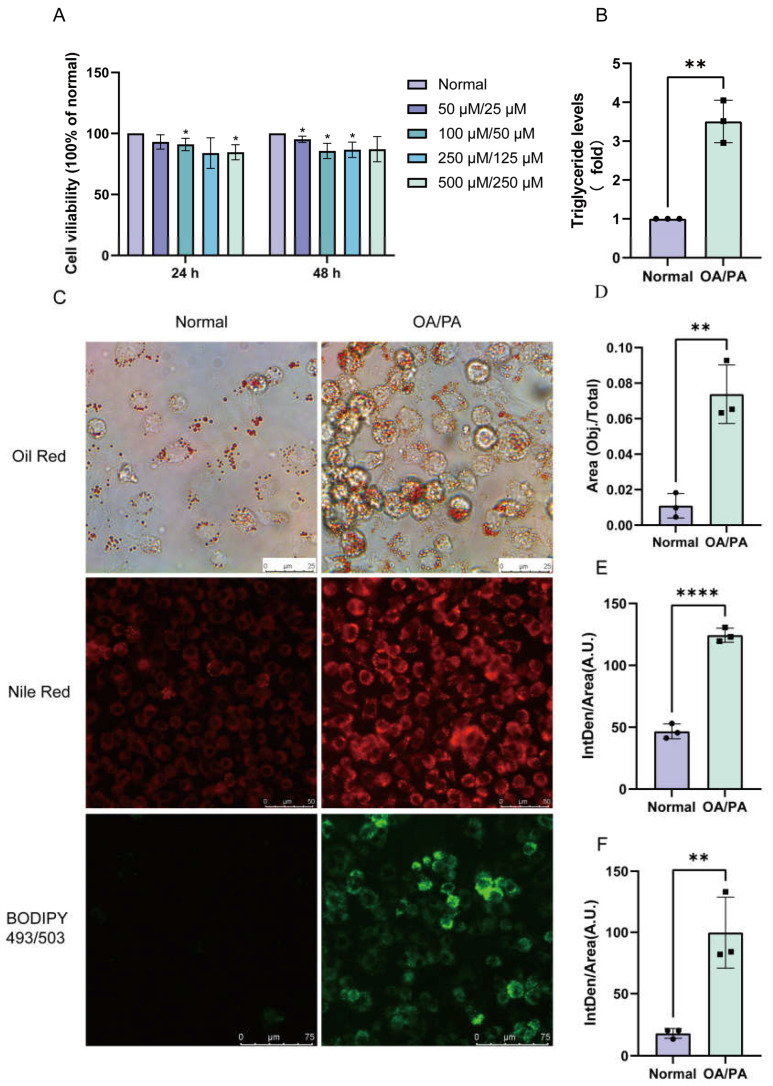
Establishment of the lipid-accumulation cell model. (**A**) The dose- and time-dependent cell survival assays of OA/PA. (**B**) OA/PA increased the TG accumulation in NCTC 1469 cells. (**C**) Representative photomicrographs for Oil Red O staining, Nile Red staining, and BODIPY 493/503. (**D**) Oil Red O staining area percentage is calculated in Figure 1C. Mean fluorescence intensity of Nile Red staining (**E**) and BODIPY 493/503 (**F**) is shown in Figure 1C. The data are presented as the mean ± SD of three independent experiments. * *p* < 0.05, ** *p* < 0.01, and **** *p* < 0.0001 vs. normal group; OA/PA, oleic acid, and palmitic acid.

To establish a cellular model of hepatic lipid accumulation, we first investigated the dose (50 μM/25 μM, 100 μM/50 μM, 250 μM/125 μM, and 500 μM/250 μM) and time-dependent (24 and 48 h) cell survival assays of OA/PA (Figure 1A). Next, 500 µM OA in 0.1% *w*/*v* FFA-free bovine serum albumin (BSA) and 250 µM PA in 0.1% *v*/*v* methyl alcohol were selected for the induction of NCTC 1469 cells for 24 h to establish the NAFLD cell model. We monitored the intracellular lipid droplets by staining them with Oil Red O, Nile Red, and BODIPY 493/503. As shown in Figure 1C–F, lipid accumulation was significantly increased in the OA/PA-induced group compared to the normal group, demonstrating the successful construction of our lipid accumulation model. Furthermore, the triglyceride assays showed an increase in the TG levels in the model group in comparison to the normal group (Figure 1B).

### 2.2. De Novo Synthesis Was Inhibited in OA/PA-Induced Lipid-Accumulation Cell Models

**Figure 2:**
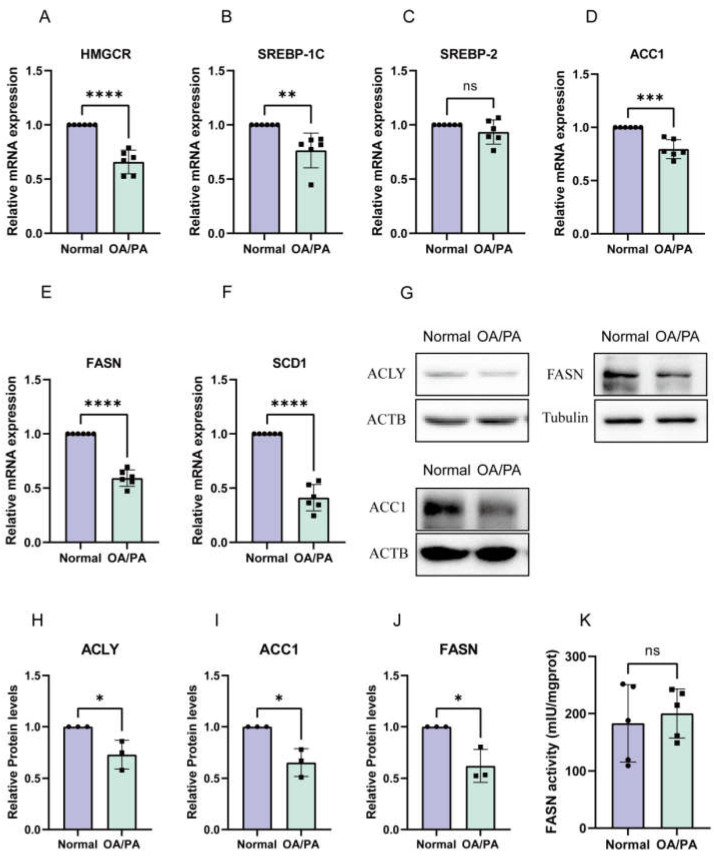
OA/PA-induced decreased expression of genes associated with de novo synthesis in hepatocytes. (**A**–**F**) OA/PA-induced changes in HMGCR, SREBP-1C, ACC1, FASN, and SCD1 mRNA expression in NCTC 1469 cells. The data are presented as the mean ± SD of six independent experiments. ** *p* < 0.01, *** *p* < 0.001, and **** *p* < 0.0001 vs. normal group. (**G**–**J**) OA/PA-induced decreased protein expression of ACLY, ACC1, and FASN in NCTC 1469 cells. The data are presented as the mean ± SD of three independent experiments. * *p* < 0.05 vs. normal group. (**K**) The enzyme activity of FASN. The data are presented as the mean ± SD of three independent experiments.

To investigate the underlying mechanism, we measured the mRNA expression of genes involved in lipid synthesis. Sterol regulatory element-binding protein 2 (SREBP-2) and anti-3-hydroxy-3-methylglutaryl coenzyme A reductase (HMGCR) are both cholesterol-synthesis-related genes; SREBP-1C, ACC1, FASN, and SCD1 are triglyceride-synthesis-related genes; and their mRNA levels were reduced (Figure 2A–F). We then characterised the expression of proteins associated with DNL in NCTC 1469 cells. The results showed that the protein expression of ACLY, ACC1, and FASN was reduced in the OA/PA-induced group compared with the control group (Figure 2G–J). Finally, we detected the enzymatic activity of FASN and found that there was no significant difference in the enzymatic activity of FASN before and after OA/PA induction (Figure 2K).

### 2.3. Enhanced Absorption of FFAs in OA/PA-Induced Lipid-Accumulation Cell Models

**Figure 3:**
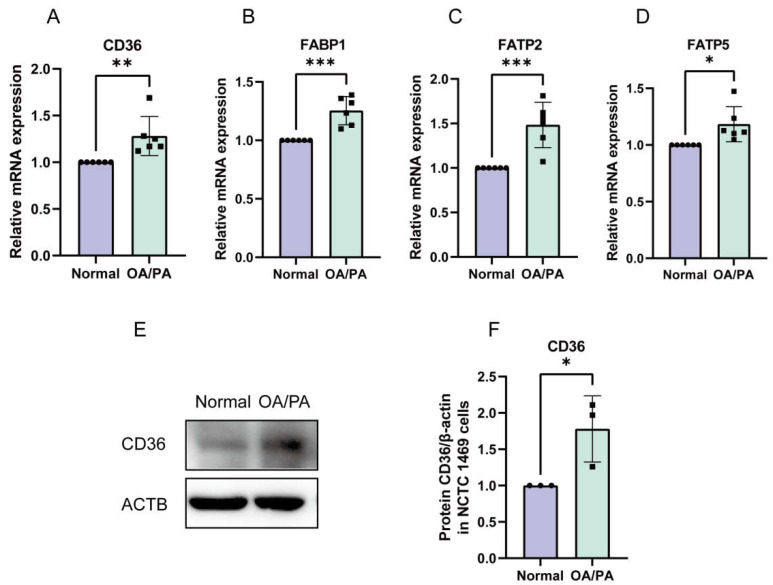
FFA uptake pathways were activated in cell models. (**A**–**D**) OA/PA-induced increased mRNA expression of CD36, FABP1, FATP2, and FATP5. The data are presented as the mean ± SD of six independent experiments. * *p* < 0.05, ** *p* < 0.01, and *** *p* < 0.001 vs. normal group. (**E**,**F**) OA/PA-induced increased protein expression of CD36. The data are presented as the mean ± SD of three independent experiments. * *p* < 0.05 vs. normal group.

To further explore the underlying mechanism, we examined the effect of FFA uptake on the lipid accumulation in hepatocytes under cell models. The results showed that the mRNA and protein levels of genes involved in FFA transport (CD36, FABP1, FATP2, and FATP5) were increased, with significant differences among cell models (Figure 3A–F).

### 2.4. Establishment of an HFD-Induced NAFLD Model

**Figure 4:**
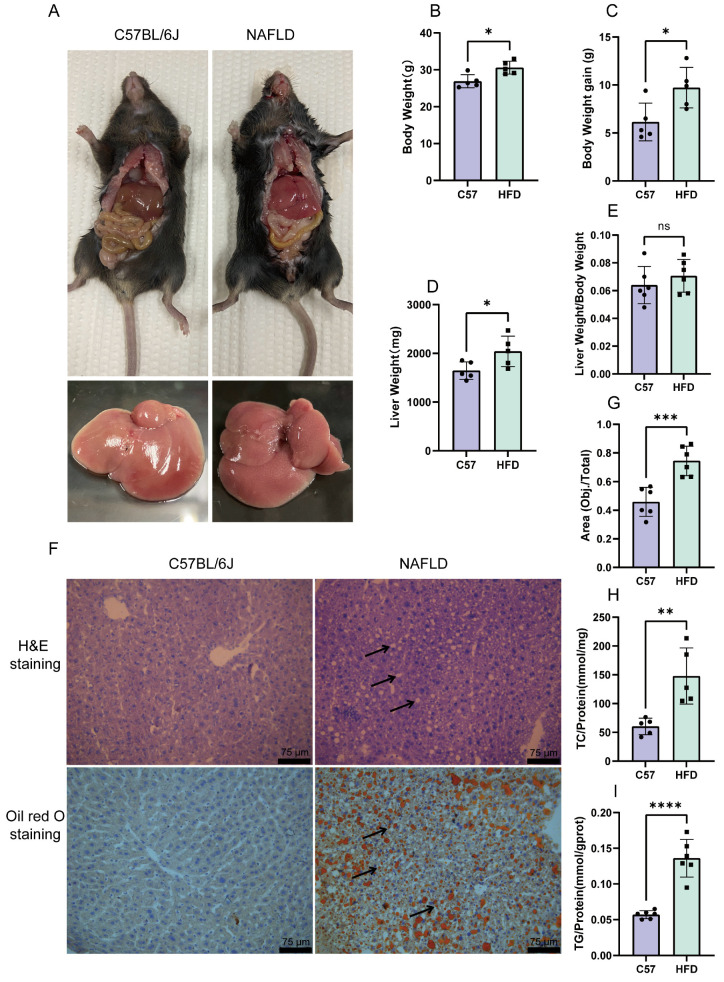
Establishment of an HFD-induced NAFLD model. Physical photographs (**A**) of mice and changes in body weight (**B**), body weight gain (**C**), liver weight (**D**), and liver weight/body weight ratio (**E**). (**F**) H&E staining and Oil Red O staining. (**G**) Oil Red O staining’s calculated area percentage shown in Figure 4F. The total cholesterol (**H**) and triglyceride (**I**) assays. The data are presented as the mean ± SD of six independent experiments. * *p* < 0.05, ** *p* < 0.01, *** *p* < 0.001, and **** *p* < 0.0001 vs. C57 group.

In the preliminary trial, after 12 weeks of consuming the HFD, the fat deposition in the liver was significantly increased in the HFD group. Compared with the C57 group, the mice in the HFD group gained weight and had grainy livers (Figure 4A–C). The liver weight increased (Figure 4D), but there was no difference in the liver weight/body weight ratio (Figure 4E). Compared with C57BL/6J mice, the hepatocytes from mice in the HFD group showed balloon-like changes under H&E staining and a large number of intracellular lipid droplets under Oil Red O staining (Figure 4F,G). Furthermore, the triglyceride and total cholesterol assays showed that TG and TC levels were increased in the model group compared to the control group (Figure 4H,I).

### 2.5. De Novo Synthesis in HFD-Induced NAFLD Model was Enhanced

**Figure 5:**
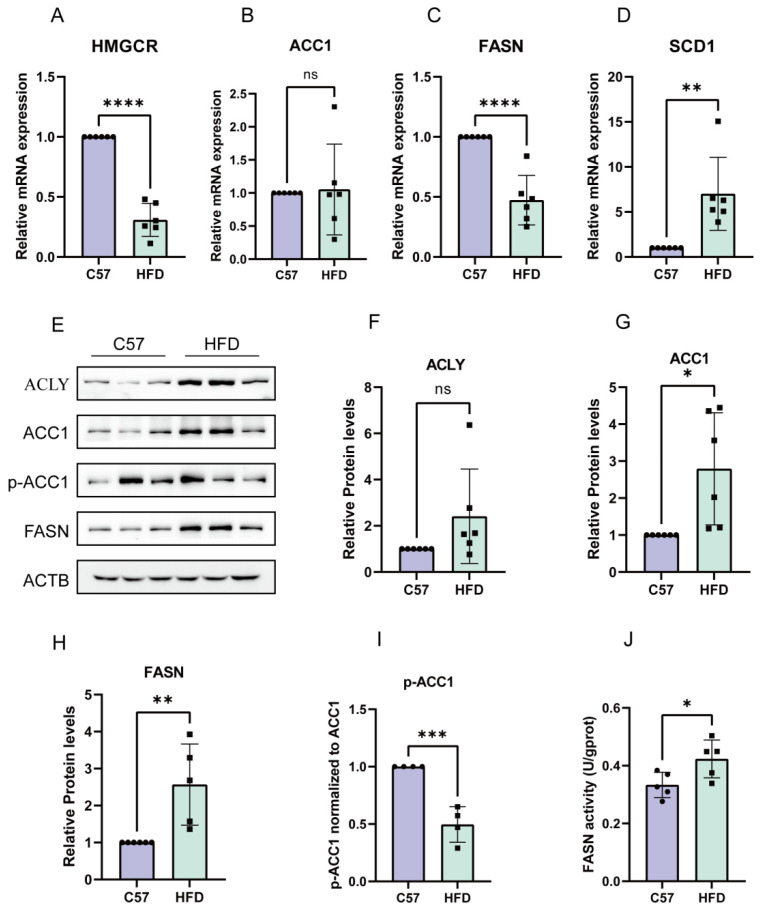
Expression of mRNA and protein of genes related to fatty acid de novo synthesis in NAFLD mice. (**A**–**D**) Altered expression levels of HMGCR, ACC1, FASN, and SCD1 mRNA in mice fed a high-fat diet. (**E**–**I**) HFD mice had significantly increased protein expression of ACLY, ACC1, p-ACC1, and FASN. (**J**) FASN activity in the liver. The data are presented as the mean ± SD of six independent experiments. * *p* < 0.05, ** *p* < 0.01, *** *p* < 0.001, and **** *p* < 0.0001 vs. C57 group.

After establishing the animal model of NAFLD, we examined the indices related to DNL. Although no obvious changes in the mRNA expression of ACC1 were observed in the HFD group compared with the control group, the mRNA expression of other genes (HMGCR and FASN) related to de novo synthesis was markedly decreased, whereas the mRNA expression of SCD1 was increased (Figure 5A–D). Subsequently, we examined the protein expression of de novo synthesis-related genes, including ACLY, ACC1, and FASN. As shown in Figure 5E–I, HFD treatment significantly increased the protein expression of ACLY, ACC1, and FASN and decreased the phosphorylation of ACC1. Finally, we measured the activity of the FASN enzyme in the liver and found that the activity of the FASN enzyme was increased in HFD group (Figure 5J).

### 2.6. Enhanced Absorption of FFAs in NAFLD Models Induced by HFD

**Figure 6:**
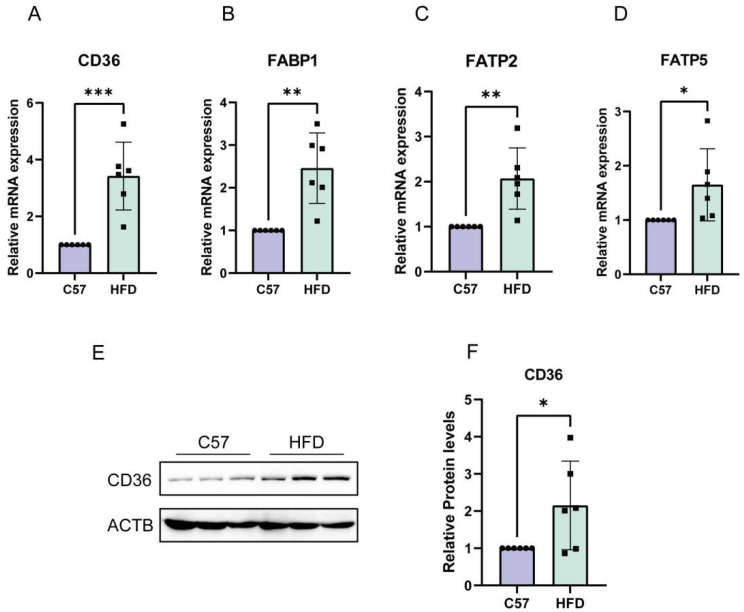
FFA uptake pathways were activated in animal models. (**A**–**D**) HFD mice had increased mRNA expression of CD36, FABP1, FATP2, and FATP5. (**E**,**F**) HFD mice had increased protein expression of CD36. The data are presented as the mean ± SD of six independent experiments. * *p* < 0.05, ** *p* < 0.01, and *** *p* < 0.001 vs. C57 group.

To further explore the underlying mechanism, we examined the effect of FFA uptake on the lipid accumulation in hepatocytes in animal models. The results showed that the mRNA and protein levels of genes involved in FFA transport (CD36, FABP1, FATP2, and FATP5) were increased, with significant differences among animal models (Figure 6A–F).

### 2.7. Serum FASN Levels Were Reduced in NAFLD Mice

**Figure 7:**
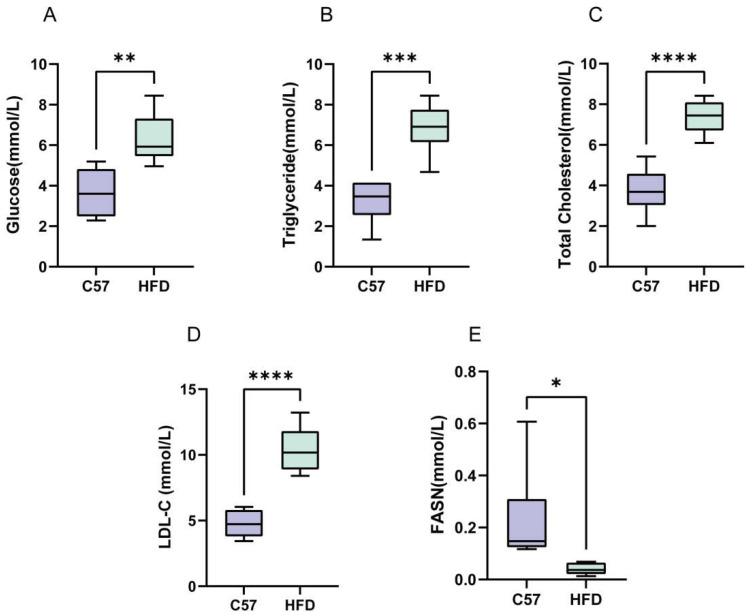
Serological markers in NAFLD mice. (**A**–**D**) The levels of fasting blood glucose, triglyceride, total cholesterol, and LDL in NAFLD-mouse-model mice. The data are presented as the mean ± SD of six independent experiments. (**E**) Serum FASN levels in NAFLD mice. * *p* < 0.05, ** *p* < 0.01, *** *p* < 0.001, and **** *p* < 0.0001 vs. C57 group.

In order to find diagnostic indicators for patients with NAFLD, 12 mice (6 C57BL/6J subjects and 6 NAFLD subjects) were selected as test subjects to determine the serum glucose, fat, and FASN levels. As shown in Figure 7A–D, the serum triglyceride, total cholesterol, LDL-c, and blood glucose levels were also significantly increased in the high-fat-model mice. Finally, we found that serum FASN levels were reduced in NAFLD mice compared to controls (Figure 7E).

## 3. Materials and Methods

### 3.1. Cells and Cell Culture

NCTC 1469 cells were obtained from Procell Life Science & Technology. Firstly, the cells were grown in Dulbecco’s Modified Eagle’s Medium (DMEM, Procell, Wuhan, China), containing 10% donor equine serum (HS, Procell, Wuhan, China). After that, the cells were cultured in a moist 37 °C incubator with 5% CO_2_. For treatment, NCTC 1469 cells were treated with or without OA/PA in 0.1% *w*/*v* bovine serum albumin (BSA) and 0.1% *v*/*v* methyl alcohol for 24 h.

### 3.2. Establishment of NAFLD Mouse Models

Six-week-old healthy male C57BL/6J mice (weighing between 20 and 21 g) were obtained from Beijing Vital River Laboratory Animal Technology Co., Ltd. The mice were initially fed adaptive diets for 1 week. Feeding conditions included adequate food and water, a comfortable temperature of 24 ± 1 °C, and a 12 h alternating light/dark cycle. The animal experimental protocols in this study were approved by the University of Chinese Academy of Sciences Animal Care and Use Committee. Mice were fed either standard chow (11.8% fat, 65.1% carbohydrates, 23.0% protein; BEIJING KEAO XIELI FEED CO., LTD; Beijing; China) or a high-fat diet (HFD), in which 40% of calories were from fat, 40% from carbohydrates, and 20% from protein (cat. no. AMLN; Dyets; Wuxi; China), and the high-fat diet was maintained for 12 weeks.

### 3.3. Cell Staining

After three washes with PBS, samples were fixed with 4% polyformaldehyde (Leagene, Beijing, China) for 15 min. The cells were then treated with 0.5% Triton X-100 for 15 min. Finally, appropriate staining was performed at room temperature. For Oil Red O staining, the cells were stained with a 2% *w*/*v* Oil Red O working solution for 60 min. Cells were stained with 2 µM Nile Red solution for 30 min for the Nile Red staining. For neutral droplet staining, the cells were stained with 4 µM BODIPY 493/503 solution for 20 min. After staining, the cells were washed three times with PBS to remove excess dye and then imaged using a light microscope at 200× magnification (Leica Microsystems, Wetzlar, Germany).

### 3.4. Evaluation of Hepatic Lipid Accumulation in Mice

After 12 weeks of high-fat diet, total cholesterol (TC) and triglyceride (TG) concentrations in mice were determined using a total cholesterol assay kit (A111-1-1; Nanjing Jiancheng Bioengineering Institute, Nanjing, China) and a triglyceride assay kit (A110-1-1; Nanjing Jiancheng Bioengineering Institute, Nanjing, China), respectively. Liver sections were stained with Oil Red O (Solarbio, Beijing, China) staining and haematoxylin and eosin (H&E; Solarbio, Beijing, China) staining according to the manufacturer’s protocol to assess hepatic steatosis in mice.

### 3.5. LDL-C Measurements

LDL-C measurements were performed using a low-density lipoprotein cholesterol (LDL-C) content assay kit (Solarbio, Beijing, China) according to the manufacturer’s instructions.

### 3.6. Glucose Metabolism Evaluation in Mice

After 12 weeks of the high-fat diet, non-fasting blood glucose and fasting blood glucose levels were monitored using a blood glucose content assay kit (Solarbio, Beijing, China) according to the manufacturer’s instructions.

### 3.7. Mouse FASN Elisa Assay

Circulating levels of FASN were monitored after 12 weeks of high-fat feeding using a mouse FASN ELISA kit (J&L Biological, Shanghai, China) according to the manufacturer’s instructions.

### 3.8. FASN Activity Assay

FASN activity assay using a fatty acid synthetase activity assay kit (Givei, Shanghai, China) according to the manufacturer’s instructions.

### 3.9. Quantitative Real-Time PCR

According to the manufacturer’s instructions, total RNA was isolated using an RNA purification kit (BS259A, Biosharp, Hefei, China). Hifair III 1st Strand cDNA Synthesis SuperMix for qPCR (11141ES60, Yeasen, Shanghai, China) was used to reverse transcribe a total of 2 μg of RNA. PCR amplification was performed using TransStart Top Green qPCR SuperMix (+Dye II) (AQ132-24; TransGen Biotech, Beijing, China). The primers used are listed in Table 1. The results were normalised to β-actin expression and calculated using the 2^−ΔΔCt^ method.

### 3.10. Western Blot Analysis

Total protein was extracted using a RIPA lysis buffer system (R0010, Solarbio Science&Technology, Beijing, China) according to the manufacturer’s protocol. A BCA Detection kit (B5000; Lablead Biotech, Beijing, China) was used to measure the protein concentrations of the extracted proteins. A total of 30 µg of proteins (for each lane) were loaded into cell lysates, separated by 10% SDS-PAGE, and then transferred to PVDF membranes. Primary antibodies were used for overnight incubation at 4 °C. Primary antibodies against FASN (3180s) and β-actin (4970) were purchased from Cell Signaling Technology (Danvers, MA, USA); primary antibodies against ACLY (67166-1-Ig) and ACC1 (67373-1-Ig) were purchased from Proteintech Group (Chicago, IL, USA); and primary antibodies against CD36 (bs-8873R) were purchased from Bioss (Beijing, China). Secondary goat anti-rabbit IgG (H + L)-HRP (S0101) and goat anti-mouse IgG (H + L)-HRP (S0100) were purchased from Lablead Biotech (Beijing, China). Protein bands were visualised using an ECL kit (170-5060; Bio-Rad, Hercules, CA, USA). To quantify the expression of the proteins, the intensity of the bands was normalised to the corresponding β-actin control, and the bands of the phosphoproteins were normalised to the total amount of each specific protein. ImageJ version 6 (National Institutes of Health, Bethesda, MD, USA) was used for densitometric analysis.

### 3.11. Statistical Analysis

Data are presented as the mean ± SD of at least three independent replicates. Statistical analysis was performed using GraphPad Prism version 9.0 (GraphPad Software, Inc., La Jolla, CA, USA). Statistical differences between the two groups were determined using a two-tailed Student’s *t*-test. A *p* < 0.05 was considered to indicate a statistically significant difference.

## 4. Discussion

**Figure 8:**
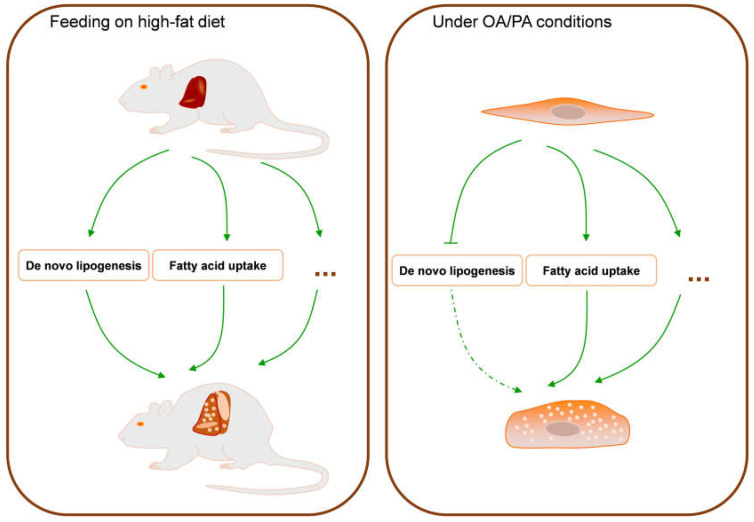
Hypothetical flowchart of hepatocyte steatosis in two models.

In this study, both HFD-induced animal models and OA/PA-induced cellular models promoted lipid accumulation in liver cells. However, the main cause of steatosis in the hepatocytes in cellular models is FFA uptake, whereas steatosis is mainly caused by de novo synthesis and FFA uptake in animal models (Figure 8). In addition, reduced levels of FASN were found when serum indicators were analysed in the NAFLD mouse model.

There is now overwhelming evidence that fatty acids mainly enter cells by a regulated pathway rather than membrane diffusion, such as CD36-mediated endocytosis [12], fatty acid transport proteins (FATPs)’ mediated proactive transit [13], and macropinocytosis-mediated fatty acid uptake [14]. There are also some specialised small proteins such as fatty acid binding proteins (FABPs) that bind FFAs and facilitate their intracellular transport [15]. FABPs are involved in the metabolism of cellular lipids, including the uptake, transport, oxidation, synthesis, and storage of fatty acids, and in the regulation of nuclear receptors [16]. FABP1 (also known as liver-type fatty acid binding protein) is a small molecule of 14 kDa that is predominantly expressed in the liver (2–5% of the protein in the cytosol) [17]. FATP2 is a transmembrane protein with a molecular weight of 70,312 Da and 620 amino acids that is encoded by Slc27a2 (solute carrier family 27 member 2) [18]. As a very long-chain acyl-coenzyme A (CoA) synthetase (ACSVL), the activation of long-chain fatty acids (LCFAs) and the transport of LCFAs as a fatty acid transporter are two major functions of FATP2 [19]. A member of the Slc27a5 gene family, the solute carrier family 27 member 5 gene (*Slc27a5*) codes for FATP5, which is mainly expressed in the liver and kidney and functions in fatty acid transport and bile acid metabolism [20]. The function of FATP5 is, as a fatty acid transporter, to transport LCFAs. CD36 is a transmembrane protein that plays an important role in fatty acid uptake and transport. CD36 is a class B scavenger receptor that is widely expressed in various tissues and cells. There are fatty acid binding regions in its structure [21]. The expression of the above proteins was increased in both the high-fat-diet-induced animal model and the OA/PA-induced cellular model that we established, suggesting that both models can lead to hepatocyte steatosis by enhancing the fatty acid transport pathway. Therefore, the models developed in this study can both be used as alternative models for partial studies of the FFA transport mechanisms of liver fat accumulation (hepatocyte steatosis) in human diseases.

CD36 deletion in hepatocytes reduced HFD-induced hepatic steatosis, decreased hepatic fatty acid uptake, and improved whole-body insulin sensitivity [22]. In both the animal and cell models of cellular steatosis used in this study, CD36 expression levels were elevated. It was shown that the uptake of FFAs via CD36 is one of the important pathways leading to lipid accumulation in the hepatocytes. NAFLD01, a specific target of the aptamer CD36, was shown to ameliorate fat deposition in vitro through interaction with CD36 [23]. Zhang P et al. [24], in a study of 456 patients with NAFLD and 403 patients without diagnosed NAFLD, showed that serum prolactin (PRL) and hepatic prolactin receptor (PRLR) expression were significantly reduced in patients with NAFLD and negatively correlated with CD36 expression. Subsequently, PRL treatment or PRLR overexpression significantly reduced CD36 expression and lipid accumulation in an FFA-induced HepG2 cell model. This suggests that PRL may ameliorate NAFLD by inhibiting CD36. B cell lymphoma 6 (BCL6) directly binds to the promoter region of CD36 and represses CD36 transcription. Zhang H et al. [25] induced hepatic CD36 expression and exacerbated hepatic steatosis by constructing BCL6 knockout mice (BCL6-KO). They showed that BCL6 could alleviate NAFLD in mice by inhibiting CD36. Berberine (BBR) is an isoquinoline alkaloid extracted from *Coptis chinensis*. Yu M et al. [26] found that the elevation of hepatic CD36 protein levels in HFD-fed mice was significantly reversed by feeding them BBR. *Curcumin*, a polyphenol extracted from turmeric root, was found by Yan C et al. [27] to significantly reverse the relative mRNA levels of hepatic CD36 and reduce fatty acid uptake in high-fat, high-fructose (HFHFr)-diet mice fed curcumin. *Gypenoside* (GP) is a traditional Chinese medicine (TCM) extracted from plants, and Huang X et al. [28] found that GP alleviated steatohepatitis and reversed the upregulation of CD36 levels in mice fed a high-fat, high-cholesterol (HFHC) diet. It is suggested that CD36 may be a target for the treatment of NAFLD.

Hepatocyte DNL plays an important role in hepatocyte steatosis. In NAFLD patients, DNL contributes 26.1 ± 6.7% of the lipid storage resources in the liver [29]. Hepatocellular steatosis, as an in vitro model of NAFLD that is routinely induced by OA/PA, plays an important role in studying the pathogenesis of NAFLD. It was shown that after establishing a NAFLD cell model in HepG2 cells, the DNL pathway was activated in the OA/PA group compared to the normal group [30,31]. In this study, we detected the expression of genes related to fatty acid synthesis. Among them, HMGCR and SREBP-2 are cholesterol-synthesis-related genes, and SREBP-1C, ACLY, ACC1, FASN, and SCD1 are triglyceride-synthesis-related genes. However, in our induced cell model, the expression levels of genes related to fatty acid synthesis were reduced, and the enzymatic activity of FASN was not altered. Similar to our results, in the NAFLD cell model constructed using HepG2 cells and OA/PA, the key proteins for fatty acid ab initio synthesis, ACC1 and SCD1 [32], and the key proteins for fatty acid ab initio synthesis, FASN [33,34] and SREBP-1 [35] mRNAs, were decreased. In addition, FAO cells (rat hepatocellular carcinoma cells) treated with OA induction also showed decreased gene expression of SREBP-1c, HMGCR, FASN, and ACC [36]. This suggests that the in vitro model of NAFLD established by OA/PA may be less applicable when exploring mechanistic studies of DNL. This may be due to the fact that OA and PA are themselves products of DNL, and excess OA and PA may, in turn, inhibit DNL and reduce mRNA and the protein levels of genes involved in DNL [30,31,32,33,34,35]. The OA/PA-induced hepatocyte lipid-accumulation model is not applicable to the study of DNL in the pathogenesis of NAFLD. In contrast, in the high-fat-diet-induced NAFLD mouse model, the mRNA expression of HMGCR and FASN for fatty acid synthesis was decreased, while the protein expression of ACLY, ACC1, and FASN was increased, and the enzymatic activity of FASN was increased. This indicates that the transcriptional levels of genes involved in ab initio synthesis were suppressed under high-fat-diet induction, but the protein-translation process was not inhibited but was enhanced, and the enzymatic activity of FASN, a key DNL enzyme, was also enhanced. It is suggested that a high-fat diet may promote the DNL process by enhancing the protein-translation process and increasing FASN enzyme activity. This suggests that the high-fat-diet-induced NAFLD mouse model is also applicable to mechanistic studies related to ab initio synthesis. In our study, the high-fat-diet regimen in the NAFLD animal model contained 22% fructose. Fructose is almost exclusively metabolised by the liver, and, therefore, ingested fructose is directed to the liver and mostly metabolised into triglycerides by DNL [2,37]. A study of sugary drinks in 94 healthy men showed that drinks sweetened with fructose and sucrose (glucose and fructose combined), but not glucose, increased the liver’s ability to produce lipids [38]. Fructose reduces fatty acid oxidation (FAO) [39]; the likely reason for this is that increased malonyl coenzyme A (an intermediate in lipogenesis) after DNL enhancement inhibits the FAO rate-limiting enzyme CPT1 alpha. Therefore, whether the addition of appropriate concentrations of fructose in the case of OA/PA induction can establish a cellular model closer to the animal model needs to be further investigated.

Experimental results in animal models suggest that FASN may also be a target for the treatment of NAFLD. There is a noticeable variable in the prevalence of NAFLD between populations and in the phenotypic expression of its severity. These differences are attributable to numerous factors including metabolic co-morbidities, the microbiome, and environmental and genetic/epigenetic factors [40]. Fatty acid synthase (FASN; E.C.2.3.1.85) is a rate-limiting enzyme that catalyses the synthesis of long-chain fatty acids in vivo. FASN is a key enzyme in the synthesis of endogenous fatty acids. In the presence of NADPH, FASN can catalyse the synthesis of acetyl-CoA and malonyl-CoA into PA [41]. Betulinic acid (BA) is a naturally occurring plant-derived pentacyclic triterpenoid that was shown to play an important role in improving metabolism. Studies showed that BA can inhibit FASN expression and alleviate lipid accumulation in the hepatocytes by inhibiting the transcription of Yin Yang 1, a GLI-Krüppel zinc finger protein, in vitro and in vivo [42]. Snx8 acts as a binding chaperone for FASN, directly binding to FASN and promoting its ubiquitylation and subsequent degradation. It was shown that the steatosis and lipogenic pathways were significantly exacerbated in the livers of Snx8 knockout mice, whereas overexpression suppressed high-fat, high-cholesterol (HFHC)-diet-induced hepatic steatosis [43]. TVB-2640 is a selective, potent, and reversible inhibitor of human FASN enzyme activity [44]. Therefore, therapeutic agents targeting FASN, such as TVB-2640 (FASN inhibitor), entered clinical trials to treat NAFLD. The administration of TVB-2640, a fatty acid synthase inhibitor, resulted in a ≥30% reduction in liver fat from baseline in 61% of patients [44]. Furthermore, our results suggest that in NAFLD the mRNA expression level of FASN is opposite to its protein level. Therefore, targeting the translation and enzymatic activity of FASN proteins for therapeutic purposes is practical and effective. Combined with CD36, discussed in the previous section as a target to mitigate FFA uptake, a combination therapy targeting FASN and CD36 may be a more desirable therapeutic target.

In the high-fat-diet-model group, serum glucose and lipids were elevated compared to the C57 group, and the differences were significant. This suggests that the high-fat-diet mouse model can mimic the hyperlipidemic characteristics of NAFLD patients. In addition, we found that the serum levels of FASN were reduced in serum for the animal model. This suggests that reduced serum levels of FASN may be a concomitant clinical manifestation of NAFLD; therefore, it is speculated that the serum levels of FASN may be used as an auxiliary indicator for the clinical diagnosis of NAFLD.

## 5. Conclusions

Both the OA/PA-induced cellular model and the HFD-induced animal model successfully simulated hepatocyte lipid accumulation. The cause of the hepatocyte lipid accumulation in the cellular model is the uptake of FFAs by hepatocytes rather than ab initio synthesis, whereas the cause of the hepatocyte lipid accumulation in the animal model includes the uptake and ab initio synthesis of FFAs by hepatocytes. This cellular model may be less applicable to studies investigating the mechanisms linking ab initio synthesis to steatosis or lipid accumulation. Meanwhile, we found that the expression of CD36 was increased in both cellular and animal models. In animal models, the transcriptional level of FASN was decreased, while the protein level and the enzymatic activity of FASN were increased. This suggests that CD36 and FASN can be used as therapeutic targets for NAFLD and that studies targeting FASN should focus on the level of the regulation of translation and the enzyme activity of FASN. As CD36 and FASN represent the fatty acid uptake pathway and the ab initio synthesis pathway, respectively, combined therapy targeting them may be more effective. In addition, we found decreased serum levels of FASN in an animal disease model of NAFLD, suggesting that the serum levels of FASN could be used as one of the indicators to aid in the diagnosis of NAFLD.

## Figures and Tables

**Table 1:** Primers for RT-qPCR.

Gene Symbol	NCBI Reference Sequence	Forward Primer (5′→3′)	Reverse Primer (5′→3′)
HMGCR	NM_008255.2	CTTGTGGAATGCCTTGTGATTG	AGCCGAAGCAGCACATGAT
SREBP-1C	NM_011480.4	GTGAGCCTGACAAGCAATCA	GGTGCCTACAGAGCAAGAG
SREBP-2	NM_033218.1	GCGTTCTGGAGACCATGGA	ACAAAGTTGCTCTGAAAACAAATCA
ACC1	NM_133360.3	GAGGTACCGAAGTGGCATCC	GTGACCTGAGCGTGGGAGAA
FASN	NM_007988.3	GGTGTGGTGGGTTTGGTGAATTGT	TCACGAGGTCATGCTTTAGCACCT
SCD1	NM_009127.4	TTCTTCTCTCACGTGGGTTG	CGGGCTTGTAGTACCTCCTC
CD36	NM_007643.4	ATGGGCTGTGATCGGAACTG	TTTGCCACGTCATCTGGGTTT
FABP1	NM_017399.5	AGTCGTCAAGCTGGAAGGTGACAA	GACAATGTCGCCCAATGTCATGGT
FATP2	NM_011978.2	ACACACCGCAGAAACCAAATGACC	TGCCTTCAGTGGATGCGTAGAACT
FATP5	NM_009512.2	TGTAACGTCCCTGAGCAACCAGAA	ATTCCCAGATCCGAATGGGACCAA
β-actin	NM_007393.5	GATCTGGCACCACACCTTCT	GGGGTGTTGAAGGTCTCAAA

## Data Availability

The data presented in this study are available on request from the corresponding author.

## Sample Availability

Samples of the compounds are available from the authors.
